# Missed Opportunities for the Diagnosis of Colorectal Cancer

**DOI:** 10.1155/2015/285096

**Published:** 2015-10-04

**Authors:** Laura A. Siminoff, Heather L. Rogers, Sonja Harris-Haywood

**Affiliations:** ^1^College of Public Health, Temple University, Philadelphia, PA 19122, USA; ^2^Department of Social and Behavioral Health, Virginia Commonwealth University, Richmond, VA 23284, USA; ^3^Department of Methodology and Experimental Psychology, University of Deusto, Bilbao, 48007 Biscay, Spain; ^4^Department of Family Medicine, Case Western Reserve University, Cleveland, OH 44106, USA

## Abstract

*Objective*. To examine patient and medical characteristics which predict a missed diagnostic opportunity (MDO) for colorectal cancer (CRC). *Methods*. The sample consisted of 252 patients diagnosed with Stages 1–4 CRC who were diagnosed in the prior six months, had experienced symptoms prior to diagnosis, and were not diagnosed through routine screening. Systematic review of all medical records prior to patients' diagnosis was conducted. An MDO was defined as a clinical encounter where, even in the presence of presumptive CRC symptoms, the CRC diagnostic process is not started. *Results*. 92 patients (36.5%) experienced an MDO. Almost 80% of alternate diagnoses were other GI-GU diseases, including hemorrhoids and diverticulitis. Stomach pain, anemia, and constipation were the most common symptoms experienced by the MDO group. These symptoms, and weight loss and vomiting, were more likely to be noted in the charts of the MDO patients (*P* < 0.04). Independent risk factors for MDO included age (<50) [OR = 2.29 (1.14–4.60), *P* = 0.02] and female sex [OR = 2.19 (1.16–4.16), *P* = 0.03]. Each additional physician seen, more than doubled the MDO risk [OR = 2.05 (1.53–2.74), *P* < 0.001]. *Conclusions*. Females, younger patients, and those consulting more physicians were all more likely to experience an MDO. Continued increased training of physicians to enhance knowledge of who is vulnerable to CRC is needed in addition to an increased focus to adherence to screening recommendations.

## 1. Introduction

Colorectal cancer (CRC) is the third most common cancer in the world and fourth most common cause of cancer death worldwide [1]. In the United States (US), it is also the third most common cancer in both men and women, but the second leading cause of cancer-related deaths [2]. Early stage at diagnosis, associated with screening, is linked to better prognosis and reduced mortality from CRC [3, 4]. When identified at its earliest stages, reductions in morbidity and costs have been identified [5]. However, only 40% of CRCs are diagnosed at an early stage [6]. Therefore, recognition of CRC symptoms as early as possible coupled with prompt provision of medical attention for those who are experiencing associated symptoms is critical.

Initial patient appraisal of CRC symptoms is frequently delayed by underrecognition of symptom significance and poor follow through with physician recommendations. The literature defines patient appraisal delay as patients' failure to recognize, acknowledge, or act on symptoms [7]. A systematic review of the literature reports that appraisal delay is the main patient factor associated with lengthier patient times between experiencing a symptom and presenting it to a practitioner [8]. There is some evidence to suggest that demographic characteristics are associated with delayed medical care seeking for CRC, and these include female sex, younger age, lower education, and being a member of a minority community [9–11]. However, more research is needed to fully understand these factors.

Physicians may also delay the diagnosis by attributing CRC symptoms to other less serious causes [12]. In one study, diagnostic delay was reported to be more likely to occur when a physician did not recognize symptoms as serious [13]. This can occur with many CRC symptoms, including the cardinal symptoms of rectal bleeding, anemia, abdominal pain, weight loss, or changes in bowel habits; these symptoms are also indicative of many other, more common, but non-life threatening diseases [14, 15]. Other factors contributing to diagnostic delay may include poor physician-patient communication, resulting in physician or patient failure to explore symptoms or obtain follow-up tests or referrals to specialists [16]. Poor communication results when patients understate or minimize symptoms or when physicians do not thoroughly explore symptoms [13]. The results of these factors may result in missed diagnostic opportunities (MDOs) and unintentional delay in initiating treatment [17]. Although, to date, gender has not been shown to play a role in diagnostic delay [11] of CRC, research on patient gender in physician-patient communication suggests that female patients ask more questions, present more symptoms, and give more information in their medical history [18, 19].

There is some evidence to suggest longer CRC diagnostic delay for ethnic minorities [20], and considerable research to indicate differences in physician-patient communication based on race/ethnicity, including less provision of biomedical information, less psychosocial counseling, and less relationship building with non-White breast cancer patients [21], as well as less patient-centered communication and less positive affect towards African American patients [22].

This study examined the factors associated with MDOs. Using a sample of recently diagnosed CRC patients in two US states, data was reviewed and extracted from all medical records prior to patients' diagnoses. The study was designed to specifically test whether individuals would be more likely to have an MDO based on race and gender. Specifically, we hypothesized that MDO would be more common in non-Caucasians (potentially as a result of less patient-centered visits) and in females (possibly because providing a large amount of information increases the complexity of their clinical presentation). We also examined whether patients who had more symptoms and certain types of symptoms would be less likely to have an MDO. The importance of this to CRC is suggested by the study conducted by Astin et al. (2011) suggesting certain symptoms and multiple symptoms have increased positive predictive value [23].

## 2. Methods

### 2.1. Participants

Patients with a confirmed diagnosis of Stages 1–4 CRC were identified, between 2008 and 2010, through multiple sources to obtain complete ascertainment of eligible patient cases, at the three academic and two large private community oncology practices in Virginia and Ohio. Case finding was conducted over a two-year period by reviewing billing codes, obtaining cases from tumor board or multidisciplinary conferences, and by reviewing pathology reports of patients prospectively. All patients diagnosed in the six months prior to initial case identification were contacted and phone screened. Inclusion criteria required that patients have a diagnosis of CRC in the prior six months and have experienced symptoms prior to their diagnosis. Patients whose CRC was diagnosed as a result of routine screening were excluded. The patients' medical histories started at their diagnoses in oncology practices and were then traced back to their primary care physicians. Although case finding occurred at oncology practices, the primary care physicians who saw these patients were in private practice and fed into many healthcare systems and followed many different clinical practice guidelines for the management of CRC symptoms. This study was approved by all relevant Institutional Review Boards and all participants provided informed consent.

### 2.2. Measurement

A systematic review of all patient medical records, both electronic and paper, prior to CRC diagnosis was conducted. As in previous studies [24], a detailed review of progress notes, consultations, laboratory and pathology reports, and additional relevant data in the records were reviewed to evaluate the diagnostic process and assess it for MDOs. Three raters were trained to conduct the review under the auspices of one of the investigators (SHH), a board certified family physician. The reviews were guided by a 20-page coding manual. The coders extracted the information according to the coding manual that operationalized the definitions associated with each item; 20% were double coded with discrepancies resolved via consensus. Records were reviewed as far back as 8 years, when warranted.

The data extracted from the medical record included patient sociodemographic characteristics, the number and type of physicians seen (e.g., primary care, gastrointestinal specialists, and obstetricians/gynecologists), type of physician in which the patient first reported symptoms, the practice type (clinic, emergency department, etc.), specific symptoms noted (abdominal pain, bowel changes, diarrhea, constipation, indigestion, weight loss, blood in stool, anemia, vomiting, rectal bleeding, and nausea), tests ordered and/or performed (colonoscopy, fecal occult blood test, sigmoidoscopy, barium enema, CT Scan, Magnetic Resonance Imaging, Positron emission tomography, blood work, urine analysis, digital rectal exam, other pelvic exams, endoscopy, and hemoccult), date of first symptom noted, date of diagnosis, date of tests, date and type of definitive diagnostic test, assessment of whether or not an MDO occurred, assessment of whether watchful waiting occurred (which was categorized under MDO), list of alternate diagnoses provided (if any), and stage and staging information.

### 2.3. Main Measures

#### 2.3.1. Outcome

*Missed Diagnostic Opportunity.* The definition of MDO, adapted from the literature, is defined as a clinical encounter where, even in the presence of presumptive illness symptoms (in this case CRC), the diagnostic process is not started. Rather, alternative evaluations are initiated or an incorrect diagnosis is provided that ends the clinical evaluation process for CRC [25, 26]. All charts were rigorously reviewed and because we reviewed all visits for the CRC symptom cluster that often crossed many visits, we were able to contextualize the decisions made by physicians. Classification of an MDO, which included inappropriate watchful waiting, was reviewed by a board certified family physician (SHH). The outcome is binary—the absence of MDO refers to the commencement of a diagnostic process in response to symptoms while the presence of MDO involves the offering of alternative evaluations or incorrect diagnoses that deviate from the diagnostic process of CRC.

#### 2.3.2. Covariates

*(1) Symptoms.* Symptoms were documented if noted in the charts. Stomach or abdominal pain of any kind, bowel/stool changes, diarrhea, constipation, blood in stool, rectal bleeding, anemia/tiredness/weakness, weight loss, nausea, vomiting, and/or indigestion and additional, related symptoms, were summed to represent a total symptom count per patient.

*(2) Physician Factors.* The number and specialty of physicians consulted were recorded from the medical records.

*(3) Patient Medicodemographic Characteristics.* Age, gender, race, education, income, insurance status, and history of comorbid chronic disease were recorded. We also obtained information on prior diagnostic tests leading to CRC diagnosis including colonoscopy and other screening tests.

### 2.4. Statistical Analysis

SPSS version 20.0 was employed for analyses. Descriptive statistics were used to describe the sample, alternative diagnoses received, and time to diagnosis. Chi-square tests were used to determine significant bivariate relationships between the presence/absence of MDO and patient medicodemographic characteristics, physician characteristics, and presence/absence of specific symptoms. Independent *t*-tests were used to test the relationships between presence/absence of MDO and the three continuous variables measured: tumor size, number of total symptoms, and number of total physicians seen for symptoms prior to diagnosis. A stepwise logistic regression model was conducted to examine the independent predictors of MDO, controlling for sociodemographic characteristics. Sociodemographic characteristics shown to be significant in bivariate analyses at *P* < 0.05 were entered into the first block, followed by medical characteristics (including presence/absence of specific symptoms) and physician characteristics that were shown to be significant in bivariate analyses at *P* < 0.05. Independent variables in the logistic regression model were tested for significance using the Wald statistic at a *P* value level of 0.05. For summary purposes, Nagelkerke's *R* ^2^ (analog to the *R* ^2^ in linear regression) was used to indicate the extent of variation explained and model fit.

## 3. Results

### 3.1. Patient Medicodemographic Characteristics

Of 495 patients screened, 66% (*N* = 303) were eligible to participate, 84% (*N* = 256) consented, and 252 completed the study. The patient population consisted of 47.6% (*n* = 120) women and 53.6% (*n* = 135) Caucasian. Twenty-five percent (*n* = 64; 25.4%) were <50 years old, 47.6% (*n* = 120) had a high school education or less, 44.0% (*n* = 111) had private health insurance, and one-third of the sample (*n* = 84; 33.3%) was diagnosed with earlier stage (I and II) disease. An MDO was ascertained from the medical records of 92 patients (36.5%).  Table 1 shows the sociodemographic characteristics of CRC patients with and without an MDO.

CRC patients with an identified MDO were significantly more likely to be younger (less than age 50; *X* ^2^ = 6.74, *P* = 0.009), female (*X* ^2^ = 11.94, *P* = 0.001), and a race other than Caucasian (*X* ^2^ = 4.73, *P* = 0.03) compared to those without MDOs. The groups were similar with respect to stage at time of diagnosis, tumor size, tumor depth, and presence of metastases. Patients with an MDO had an average of 2.7 nodes positive for cancer at diagnosis compared to 2.0 for those without an MDO and this difference was marginally significant (*P* = 0.05).

Table 2 shows the patient and physician characteristics of the sample. Over half of the sample (57.1%; *n* = 144) reported having a comorbid chronic condition at the time of diagnosis (e.g., hypertension, diabetes, and cardiac conditions). Although comorbidities were common in this sample, having at least one comorbidity was not associated with an MDO. A minority of patients (18.3%; *n* = 46) had a colonoscopy or other CRC screening tests (18.9%; *n* = 47) prior to receiving the diagnostic tests that led to the CRC diagnosis. Neither of these factors was associated with MDO.

### 3.2. Symptoms

The average participant reported their symptoms to two physicians at separate visits at outpatient primary care practices, emergency rooms (ER), and/or urgent care centers (range 0–10). Those patients with MDOs reported their symptoms to significantly more physicians than those who did not (3.8 versus 2.4 physicians; *t* = 7.00, *P* < 0.001). Sixty-eight percent (*n* = 171; 67.9%) of patients first communicated their symptoms to a primary care physician (PCP) and 21.0% (*n* = 53) in the ER. Type of physician seen first was not associated with MDO. Patients with more symptoms were more likely to experience an MDO (4.7 versus 3.7; *t* = 3.29; *P* = 0.001) (see Table 2). Compared to those without an MDO, those with an MDO were significantly more likely to have the following symptoms noted in their medical records: stomach pain (*X* ^2^ = 10.20, *P* = 0.001), anemia (*X* ^2^ = 8.11, *P* = 0.004), constipation (*X* ^2^ = 5.04, *P* = 0.03), weight loss (*X* ^2^ = 4.40, *P* = 0.04), and vomiting (*X* ^2^ = 4.19, *P* = 0.04) (see Table 3). No differences between groups were noted with respect to other symptoms such as blood in stool and change in bowel habits. The percentage of individuals with each symptom who also experienced rectal bleeding was 35.3% of those with stomach pain, 24.7% of those with anemia, 34.7% of those with constipation, 32.6% of those with weight loss, and 20.5% of those with vomiting. Rectal bleeding was associated with anemia (*X* ^2^ = 5.70, *P* = 0.017) and vomiting (*X* ^2^ = 4.20, *P* = 0.04) such that individuals with anemia charted in their medical records were less likely to have rectal bleeding charted than those without anemia (24.7% versus 39.4%). Similarly, those with vomiting were less likely to have rectal bleeding reported (20.5% versus 36.5%).

### 3.3. Missed Diagnostic Opportunity, Alternative Diagnoses, and Time to Diagnosis

Diagnostic delay was defined based on the recommended refinements to The General Model of Total Patient Delay [27] proposed by Andersen and colleagues (1995) [28] stemming from earlier work by Safer and colleagues (1979) [29]. Diagnostic delay was defined as the time in months from the date of the first consultation with a healthcare provider for symptoms to the date of diagnosis. Overall, the average diagnostic delay was 4.7 months (SD = 8.2).

Almost 80% of patients with an MDO were diagnosed with an alternate GI-GU disease (*n* = 73; 79.3%), including hemorrhoids (*n* = 26), diverticulitis (*n* = 15), urinary tract infection (UTI, *n* = 9), colitis/gastritis (*n* = 7), gastroesophageal reflux disease (GERD, *n* = 6), irritable bowel syndrome (IBS, *n* = 5), and/or gastroenteritis (*n* = 5) (see Table 3). All of the individuals with alternate diagnoses of UTI were women (100%, *n* = 9), two out of three of those diagnosed with diverticulitis were women (67%, *n* = 15), and the majority of those diagnosed with hemorrhoids (58%; *n* = 15) were women. Approximately one out of four received a non-GI-GU diagnosis (*n* = 21; 22.8%). For 26 patients (28.3%), the review of the medical records indicated that the physician engaged in watchful waiting, for example, when a patient's GI symptom complaints did not result in follow-up testing and/or the only treatment recommendations involved lifestyle or dietary changes.

Mean diagnostic delay for patients without an MDO was 2.0 months (SD = 4.0), significantly shorter than 9.4 months (SD = 11.0) for patients with a documented MDO (*t* = 8.21, *P* < 0.001). Patients whose physicians did not recommend watchful waiting averaged 3.7 months (SD = 6.6), significantly shorter than 13.6 months (SD = 13.6) for those whose physicians engaged in watchful waiting (*t* = 5.40, *P* < 0.001). Patients receiving a GI-GU related alternate diagnosis averaged 9.0 months (SD = 9.9) to diagnosis while those who received a non-GI-GU diagnosis needed 10.5 months (SD = 11.5) to be diagnosed (see Table 4).

### 3.4. Logistic Regression Model Predicting Missed Diagnostic Opportunity

The final model (Table 5) demonstrates that patients who had an MDO were more than twice as likely to be less than 50 years [OR = 2.29 (1.14–4.60), *P* = 0.02] and female [OR = 2.19 (1.16–4.16), *P* = 0.03]. For each physician seen, the likelihood of MDO more than doubled [OR = 2.05 (1.53–2.74), *P* < 0.001]. Number or type of symptoms was not independently predictive of MDO. Nagelkerke's *R* ^2^ of 0.36 indicates a moderate relationship between the set of predictors in the model and MDO.

## 4. Discussion

Routine screening is important for early diagnosis of CRC. However, many Americans are noncompliant with screening guidelines and screening is not perfect; only 65% of Americans, age 50 and older, have ever undergone any type of screening test for CRC [30]. Interestingly, screening compliance rates are similar in universal care and/or socialized health care systems [31]. For instance, only 55–60% of individuals aged 60 invited to participate in the Bowel Cancer Screening Programme in England returned their first FOBT [32]. Recent studies also estimate that colonoscopy may miss detecting lesions [33]. Moreover, routine testing is not recommended for those under 50 years of age; therefore, symptom recognition remains an important component of early detection and treatment. We found evidence that being younger (<50) and female, as well as being assessed by more than one physician, were independent risk factors for MDO. Furthermore, over the course of diagnosis, stomach pain, anemia, and constipation were more frequently documented in the group of patients with MDO. These, of course, are symptoms common to other illnesses and anemia is especially prevalent in menstruating women and associated with gynecological ailments. Lastly, almost 80% of misdiagnoses were alternate GI-GU diseases, most commonly, hemorrhoids and diverticulitis, and that diagnosis with a non-GI-GU disease was associated with even longer diagnostic delay. Individuals with an MDO experienced nearly double the time to diagnosis, and patients whose physicians chose watchful waiting experienced triple the time.

Few studies have simultaneously examined patient and physician factors influencing MDO in CRC; these studies have investigated physician delay in referring patients for further investigation of symptoms. Like this study, other studies indicate that older age leads to quicker referrals and more prereferral consultations are associated with younger age and ethnic minority status [11]. This supports the results of the present study in which those under age 50 are 2.3 times more likely to experience an MDO. In univariate analyses, patients of a race other than Caucasian were more likely than Caucasians to have an MDO, although these results were not significant in the multivariable model. Our findings strongly suggest that gender plays a significant role, as 62% of the MDO group were female. Follow-up analyses found that most of the alternate diagnoses of hemorrhoids, diverticulitis, and UTIs were made in females.

Some of this study's findings are not consistent with previous work in the field, however. For instance, patients who experienced an MDO average one more documented symptom than those who did not experience an MDO. Mariscal et al. report that, as the number of symptoms presented increases, time from initial consultation to hospital admission for digestive tract cancers decreases [34]. The fact that higher number of symptoms are related to MDO but not stage suggests that patients who experienced an MDO in this study may be presenting with a more complex clinical picture. The types of symptoms these patients present with, and the time course of their multisymptom presentation, may account for this discrepancy, especially given that this sample excluded patients diagnosed through screening colonoscopy. These patients were excluded because of the study's intent to examine the role that symptom presentation plays in the diagnostic process. Our study indicates that anemia, stomach pain, and constipation were the most common symptoms of the group with MDOs. In European cohorts, iron-deficiency anemia [26, 35] and rectal bleeding [26] were the primary symptoms most often associated with MDO for CRC diagnosis. In a US study, Singh et al. also found that anemia was the most common symptom associated with MDOs for CRC diagnosis and longest referral time for an endoscopic procedure [25]. It was also associated with two-thirds of MDOs for CRC in a Veterans Administration sample [36]. Of note in our study is that, in multivariable analyses, anemia was not a significant independent predictor of MDO. Finally, a systematic review of the diagnostic value of symptoms for CRC in primary care concluded that investigation of anemia in primary care patients is warranted, regardless of whether other symptoms are present [23].

Seeing an additional physician for CRC symptoms was associated with twice the risk of MDO. Although this finding may be intuitive, as increases in seeking consultations with new physicians provides more opportunity/risk for possible MDO, to our knowledge, the importance of this factor as related to MDO and/or diagnostic delay has not been previously documented. Our sample was comprised exclusively on those individuals who experienced symptoms prior to diagnosis who were primarily diagnosed with later stage disease, whereas many other studies examine all patients diagnosed with CRC and focus on physician screening behaviors and probably drove these results. We believe it is a strength, of this study, as those diagnoses through screening are a different segment of the CRC population. Furthermore, physician training, resources, and screening guideline incentives may differ across healthcare systems and regions, thus additional investigation of the factors related to MDO, in other contexts, is warranted.

It is possible that some physicians may have made verbal diagnoses or recommendations for follow-up that were not documented in the medical records and would not have been captured using the chart review methodology. Although we compiled lists of all possible physicians seen by patients and followed-up with practices identified via documented referrals, it is possible we may have missed some, thus influencing this study's findings. Although every effort was taken to ensure that the case notes were rigorously reviewed, the timing of specific symptoms and symptom combinations in relation to the MDO was not extracted. Future studies, especially in the context of the U.S. healthcare system, and in context of the high variability in community practice, are warranted to fully explore these aspects.

In summary, our study suggests that greater training of physicians could be helpful for dispelling stereotypes about who is vulnerable to CRC. Within the population, men are only slightly more susceptible to CRC than women [1]; however, women were twice as likely to experience an MDO. Younger patients (<50 years) are also more likely to experience an MDO, giving rise to some concern that current CRC screening recommendations are leading physicians to erroneously discount the possibility of CRC in symptomatic younger patients. A recent study supports these findings, that, even lacking a family history, CRC is not insignificant [37]. Moreover, the incidence rate of CRC in this age group is increasing and now accounts for 12% of all cases of CRC [37, 38]. Given the fact that a physician has, on average, six to 18 minutes to conduct a clinical evaluation and provide follow-up recommendations (depending on the country) and that physicians feel they need more time to spend with their patients [39], future research and policy are needed to better understand and assist physicians with the demands placed on them that may result in rushed visits and ultimately an MDO.

Looking at the results as a whole, there are potential lessons for clinical practice. First, although it is important to understand the average characteristics of a patient population, the focus in the physician-patient encounter is the patient and not his/her sociodemographic group. Although 90% of new cases of CRC are in patients over 50 years of age, over 14,000 individuals less than age 50 in the US alone will be diagnosed with CRC annually [40]. Therefore, some vigilance concerning patients under the age of 50 is warranted. In this study, younger patients were more likely to have greater times to diagnosis. In addition, women were also more likely to have greater diagnostic delay, perhaps because CRC is thought to be more prevalent in men. Another source of diagnostic difficulty was demonstrated by greater time to diagnosis for patients with more symptoms. Although intuitively it would seem that more symptoms would provide more sign posts leading toward the CRC diagnosis, the greater number of symptoms, coupled with the nonspecific nature of these symptoms, seemed to add complexity rather than clarity to making a diagnosis. We posit that when patients were seen by more physicians—perhaps because the patient was not satisfied with the treatment provided by the initial physician or because they did not have easy or regular access to the health system—the discontinuity in care led to greater time to diagnosis. This is an issue that bears further exploration.

It may be that some physicians are confronting a Hobson's choice between interacting with the “whole” patient or just asking for the facts, although both pieces are likely important. This study is yet another indication that quality care takes time, skill, and the participation of both the patient and the physician.

## Figures and Tables

**Table 1:** Sociodemographic and medical characteristics by missed diagnostic opportunity (*N* = 252).

Variable	Patients who had missed diagnostic opportunity	Patients who did not have missed diagnostic opportunity	*P* value
	(*n* = 92; 36.5%)	(*n* = 160; 63.5%)	
Gender^∗∗^			
Female	57 (62.0%)	63 (39.4%)	*P* = 0.001
Race^∗^			
Caucasian	41 (44.6%)	94 (58.8%)	*P* = 0.03
Age^∗^			
<50	32 (34.8%)	32 (20.0%)	*P* = 0.009
Education			
<High school	18 (19.6%)	32 (20.0%)	*P* = 0.10
High school diploma	30 (32.6%)	40 (25.0%)	
Some college	33 (35.9%)	50 (31.3%)	
Bachelor's degree and beyond	11 (12.0%)	37 (23.1%)	
Declined to answer/do not know	0 (0.0%)	1 (0.6%)	
Income			
<$30,000	44 (47.8%)	61 (23.2%)	*P* = 0.22
$30 K–$75 K	28 (30.4%)	44 (27.5%)	
>$75 K	18 (19.6%)	45 (28.1%)	
Declined to answer/do not know	2 (2.3%)	10 (6.3%)	
Health insurance			
Private	41 (44.6%)	70 (43.8%)	*P* = 0.71
Medicare	28 (30.4%)	43 (26.9%)	
Medicaid, state insurance, or uninsured	23 (25.0%)	47 (29.4%)	
Stage			
1-2	25 (27.2%)	59 (36.9%)	*P* = 0.06
3-4	65 (70.7%)	101 (63.1%)	
Unknown	2 (2.2%)	0	
Tumor size	5.1 (2.1)	4.8 (2.0)	*P* = 0.14

^∗^ *P* < 0.05, ^∗∗^ *P* < 0.01.

**Table 2:** Patient and physician factors associated with missed diagnostic opportunity.

Variable	Patients who had missed diagnostic opportunity	Patients who did not have missed diagnostic opportunity	*P* value
	(*n* = 92)	(*n* = 160)	
Presence of comorbid condition	57 (62.0%)	87 (54.4%)	*P* = 0.24
Colonoscopy prior to the one leading to CRC diagnosis	19 (20.7%)	27 (16.9%)	*P* = 0.46
Other CRC screening tests prior to one leading to diagnosis	14 (15.4%)	33 (20.9%)	*P* = 0.29
Number of symptoms^∗∗^	4.7 (2.6)	3.7 (2.2)	*P* = 0.001
Mean number of physicians seen^∗∗∗^	3.8 (1.8)	2.4 (0.9)	*P* < 0.001
Type of physician first seen			
Primary care physician	64 (69.6%)	107 (66.9%)	*P* = 0.81
Emergency room physician	21 (22.8%)	32 (20.0%)	
Other (specialist, nurse, etc.)	7 (7.6%)	21 (13.1%)	

^∗∗^ *P* < 0.01, ^∗∗∗^ *P* < 0.001.

**Table 3:** Common chart-documented symptoms of patients with a missed diagnostic opportunity (ordered by most frequently documented in this group).

Symptom	Patients who had a missed diagnostic opportunity	Patients who did not have missed diagnostic opportunity	*P* value
	(*n* = 92)	(*n* = 160)	
Stomach pain^∗∗^	65 (70.7%)	80 (50.0%)	*P* = 0.001
Anemia^∗∗^	46 (50.0%)	51 (31.9%)	*P* = 0.004
Constipation^∗^	43 (46.7%)	52 (32.5%)	*P* = 0.03
Blood in stool	42 (45.7%)	75 (46.9%)	*P* = 0.85
Diarrhea	40 (43.5%)	52 (32.5%)	*P* = 0.08
Weight loss^∗^	39 (42.4%)	47 (29.4%)	*P* = 0.04
Change in bowels/stool	37 (40.2%)	52 (32.5%)	*P* = 0.22
Indigestion	37 (40.2%)	46 (28.7%)	*P* = 0.06
Rectal bleeding	33 (35.9%)	52 (32.5%)	*P* = 0.59
Nausea	29 (31.5%)	34 (21.2%)	*P* = 0.07
Vomiting^∗^	22 (23.9%)	22 (13.8%)	*P* = 0.04

^∗^ *P* < 0.05, ^∗∗^ *P* < 0.01.

**Table 4:** Alternate diagnoses recorded in medical chart for symptoms indicative of CRC (*n* = 92).

Alternate diagnosis^+^	*n*	Mean time to diagnosis in months	Median time to diagnosis in months
No diagnosis, missed opportunity/watchful waiting	26	13.6 (13.6)	9.2

GI-GU disease	73	9.0 (9.9)	6.3
Hemorrhoids	26		
Diverticulitis	15		
Urinary tract infection (UTI)	9		
Colitis/Gastritis	7		
Gastroesophageal reflux disease (GERD)	6		
Irritable bowel syndrome (IBS)	5		
Gastroenteritis	5		
Kidney stones	4		
OB/GYN disease (cyst, neoplasm, fibroids)	3		
Pregnancy	2		
Chronic kidney disease	2		
Small bowel disease (cancer, obstruction)	2		
Gallbladder	2		
Crohn's disease	1		
Chronic dysphagia	1		
Chronic constipation	1		
Hernia	1		
Dyspepsia	1		
*H. pylori *	1		
Appendicitis	1		
Prostatitis	1		
Anal fissure	1		
Syphilis	1		
HIV infection	1		

Non-GI-GU diagnoses	21	10.5 (11.5)	9.3
Cardiovascular	7		
Respiratory	6		
Back/skeletal	5		
Side effects of medications	4		
Osteoarthritis	2		
Fibromyalgia	1		
Insomnia	1		

^+^Some patients had more than one alternate diagnosis; 11 had both GI-GU and non-GI-GU alternate diagnoses. Because of this no tests of significance were performed.

**Table 5:** Multivariate model examining predictors of missed diagnostic opportunity.

Variable	*β*	Wald	Odds ratio (95% confidence interval)	*P* value
Gender: female^∗^	0.79	5.79	2.19 (1.16–4.16)	*P* = 0.02
Race: African American	0.31	0.96	1.37 (0.73–2.57)	*P* = 0.36
Age < 50^∗^	0.83	5.41	2.29 (1.14–4.60)	*P* = 0.02
Number of physicians seen^∗∗∗^	0.72	23.31	2.05 (1.53–2.74)	*P* < 0.001
Number of symptoms experienced	0.07	1.02	1.07 (0.94–1.23)	*P* = 0.31
Stomach pain	0.54	2.34	1.07 (0.86–3.41)	*P* = 0.26
Anemia	0.11	0.11	1.12 (0.58–2.17)	*P* = 0.74
Constipation	0.17	0.22	1.18 (0.59–2.35)	*P* = 0.64
Weight loss	0.17	0.23	1.18 (0.60–2.33)	*P* = 0.63
Vomiting	−0.26	0.32	0.77 (0.32–1.87)	*P* = 0.57

^∗^ *P* < 0.05, ^∗∗∗^ *P* < 0.001.
